# Fair enough? Decreased equity of dyadic coping across the transition to parenthood associated with depression of first-time parents

**DOI:** 10.1371/journal.pone.0227342

**Published:** 2020-02-19

**Authors:** Fabienne Meier, Anne Milek, Valentina Rauch-Anderegg, Christelle Benz-Fragnière, Jan Willem Nieuwenboom, Holger Schmid, W. Kim Halford, Guy Bodenmann

**Affiliations:** 1 Department of Psychology, University of Zurich, Zurich, Switzerland; 2 Department of Psychology, University of Münster, Muenster, Germany; 3 Massachusetts General Hospital, Harvard Medical School, Boston, Massachusetts, United States of America; 4 Institute of Social Work and Health, University of Applied Sciences and Arts Northwestern Switzerland, Olten, Switzerland; 5 School of Psychology, University of Queensland, Brisbane, Australia; Erasmus Medical Center, NETHERLANDS

## Abstract

The transition to parenthood (TTP) is a stressful life event for most couples. Therefore, the way both partners jointly cope with stress (i.e., dyadic coping) is important for the prevention of individual adjustment problems (e.g., depression). For dyadic coping to be effective in reducing depressive symptoms, efforts of both partners should be equal. However, many couples experience a decrease of equity in task division within the domestic sphere across the TTP. The current study investigates the equity of a specific skill within the ‘relationship sphere’, because similarly to a decreased equity in household and childcare, a decreased equity of dyadic coping is likely to be associated with poorer individual adjustment. We collected longitudinal self-report data on dyadic coping and depressive symptoms from 104 mixed-gender first-time parents (n = 208 individuals) from pregnancy until 40 weeks postpartum. We created an equity score for men and women that measured their perceived difference between received and provided dyadic coping. On average, women reported providing more and receiving less dyadic coping than men. While both genders agreed on this distribution, men did perceive a higher equity of dyadic coping than women. Furthermore, the decrease of equity perceived by women across TTP was not visible in men. In line with our assumptions based on the equity theory, perceived equity of dyadic coping was associated with depressive symptoms in a curvilinear manner: Decreases in women’s perceived equity in either direction (over- or underbenefit) were associated with more depressive symptoms in women *and their male partners*. This association was found above and beyond the beneficial effect of dyadic coping itself. This implies that not only how well partners support each other in times of stress, but also how equal both partners’ efforts are, is important for their individual adjustment across TTP.

## Introduction

The transition to parenthood (TTP) brings fulfillment and joy to new parents [1–4], but also high levels of parental stress [5] and depressive symptoms [6]. The high risk for depression of mothers and fathers across TTP [7] is of particular interest as parental depression negatively affects child development during the first year after birth [8] up until the age of 18 years [9]. One factor that has been associated with psychological distress across TTP is the perception of decreasing equity in the domestic sphere, with increasingly unequal distribution of household or child care tasks between men and women [10,11]. In mixed-gender couples, women’s work load in the domestic sphere tends to increase more across TTP than men’s work load [12,13]. This gender-specific change across TTP has been described as the traditionalization shift [14]. The current study analyses whether such a shift is also visible in a specific behavior within the ‘relationship sphere’, indicated by how well partners support each other in times of stress (i.e., dyadic coping).

### Equity of dyadic coping and distress across the TTP

The general perception of equity of both partners’ inputs into the relationship was found to be associated with lower distress in newlyweds before birth of their first child [15]. However, specific behaviors such as supporting each other in times of stress (i.e., dyadic coping) were never analyzed in terms of equity across TTP even though we know from community samples that specific support behavior is affected by gender role expectations [16], which become more salient across TTP. We argue that besides domestic tasks [10], relationship tasks such as dyadic coping are relevant for parents’ depressive symptomatology as well. We therefore analyze perceived equity of dyadic coping and how equal men and women perceive each other’s dyadic coping efforts (i.e., perceived equity of dyadic coping) across TTP. Similar to domestic tasks, we expect women to provide more dyadic coping than they receive and that this inequity of dyadic coping increases across TTP, especially in women. In accordance with findings in community samples [17], we expect perceived inequity of dyadic coping to be associated with depressive symptoms.

Depressive symptoms are common in women and men transitioning to parenthood [6]. Prevalence varies across countries [7] with a mean-estimate of around 10% in men and 20% in women during the first year postpartum [18]. One of the key protective factors of depressive symptoms across TTP is social support [19]. Across various complex definitions, the common defining aspect of social support are supportive resources that are given to a recipient with whom the provider is in a social relationship [20]. The most common source of social support is the partner with whom the recipient is in an intimate relationship [21]. Social support by the partner (which has also been called spousal support) is associated with parental health and well-being across TTP [22,23]. A special form of social support, which has been specifically conceptualized for close relationships, is dyadic coping [24]. While spousal support entails a wide variety of behaviors such as praising the partner [25], confiding [26], or being encouraging [27], dyadic coping focuses on behaviors of one partner to support the other partner when he or she is stressed [24]. This is of particular interest for depressive symptomatology, as stress was found to be one of the main contributors to psychopathology in general [28] and to depression in particular [29]. Based on the systemic-transactional model of stress [30], stress experienced by one partner affects the other partner directly or indirectly. Therefore, more positive dyadic coping (supportive dyadic coping: e.g., listening, showing empathy, and calming the partner down; delegated dyadic coping: e.g., taking over tasks the partner normally does in order to alleviate stress) and less negative dyadic coping (e.g., not taking the stress seriously, half-hearted support or blaming the partner for his/her coping with stress) are associated with lower psychological distress of both partners [31]. Dyadic coping was associated with lower depressive symptoms in community samples [32], in couples facing depression [33], and in pregnant women and their partners [34]. We therefore expect dyadic coping to play an important role across the TTP as it is a period of high stress for both partners [5] and both partners’ psychological distress is highly interdependent [35].

Similar to concepts of social support [36], the concept of dyadic coping assumed from the very beginning that dyadic coping will only be beneficial if both partners invest equal efforts to support each other in times of stress [37]. If we understand dyadic coping as a resource exchanged in close relationships, partners will aim for a just exchange of this resource between them [38]. Consistent with the basic normative assumption of Western societies that invested efforts should be repaid [39–41], partners will be satisfied with the exchange when they perceive equity and experience negative feelings when they perceive inequity. Equity is defined as similarity of inputs (i.e., provided dyadic coping) and outputs (i.e., received dyadic coping) of two interacting people as subjectively perceived by one person [38]. Studies analyzing equity of dyadic coping found that in community samples, lower perceived differences between provided and received dyadic coping (i.e., equity of dyadic coping) as perceived by women–not men—was associated with general health reported by men and women [17]. Accordingly, we expect perceived equity to be associated with less depressive symptoms beyond negative associations with dyadic coping across TTP. At the same time, gender differences in the equity of dyadic coping are expected, as illustrated in the following.

### Gender differences across the TTP

A recent review showed that in Western mixed-gender samples, men and women report that women provide more dyadic coping than men [31]. While these gender differences are rather small [42], they exacerbate under stress [43]. With respect to the direction of inequity we therefore expect women to perceive an underbenefit (received dyadic coping < provided dyadic coping) and men to perceive an overbenefit (received dyadic coping > provided dyadic coping). As an effect of the increased stress [5] and the traditionalization of gender roles [12–14], we expect that these perceived inequities increase across TTP with increasing underbenefits for women and overbenefits for men. Based on the equity theory, inequity will be associated with more depressive symptoms in either direction [38]. According to the social exchange theory on the other hand, individuals strive for cost-optimization (i.e., increasing outputs compared to inputs) [44]. Research on spousal support found the opposite. On days when partners reported that they had provided support (i.e., input), they reported higher self-esteem than on days when they reported receiving support (i.e., output) [45,46]. On days when they received support, they even reported more distress, unless they also reported providing support on that same day (i.e., days of reciprocal support) [47]. In line with these findings receiving less support than providing was found to be as detrimental as receiving more than providing [48]. Therefore and in accordance with the equity theory [38], we expect differences between provided and received dyadic coping to be associated with distress in a curvilinear manner. That is, the lower each person’s perceived equity (higher difference between received and provided dyadic coping) in either direction (over- or underbenefit), the higher we expect depressive symptoms to be. We examined actor and partner effects, as a previous study found equity of dyadic coping as perceived by women to be predictive of men and women’s general health in a community sample while men’s perceived equity showed no associations with general health [17]. Furthermore, there are many studies showing the beneficial effects of dyadic coping for couples’ adjustment [49,50]. Therefore, we expect an interaction between perceived equity of dyadic coping and dyadic coping itself. When a person perceives a high equity (low difference between received and provided dyadic coping) on a high level of dyadic coping (high provided and high received dyadic coping), he or she will report lower depressive symptomatology than a person who perceives a high equity on a low level of dyadic coping (low provided and low received dyadic coping).

### The current study

In the current study, mixed-gender couples, that were about to become parents for the first time, provided self-report data at two time points before birth (T1: 27^th^ week of pregnancy and T2: 32^nd^ week of pregnancy) and three time points after birth (T3: 2 weeks after birth, T4: 14 weeks after birth, and T5: 40 weeks after birth). These men and women provided information about the dyadic coping they received from their partner and the dyadic coping they provided to their partner, according to their subjective perception. For each person we created an equity index, which reflected the difference between received and provided dyadic coping as perceived by each partner. The first aim of the current study was to investigate whether men and women differed in their perception of equity. Second, we examined the course of perceived equity across TTP. We hypothesized perceived equity of dyadic coping would be stable during pregnancy and decrease after birth, when demands of infant care, sleep disturbance, and potential traditionalization shifts are impacting the couple. Third, we examined the association between perceived equity and depressive symptoms, whether there where partner effects of equity on depression, and whether dyadic coping itself moderated this association.

## Material and methods

### Participants

Participants were recruited during the third trimester of pregnancy with their first child. Couples had to be in a committed mixed-gender relationship for at least one year, had to read and speak German, and did report currently not being treated for physical or psychological illnesses. Recruitment took place with leaflets in different hospitals, gynecological practices, and pregnancy yoga courses, as well as via newspaper ads and the homepage of the institute. Each couple received study documentation and signed a written informed consent form. The Ethic Committee of the Department of Psychology of the University approved the study (approval number: 18-12-2013).

One hundred and four couples (n = 208 individuals) aged between 26 and 63 years at T1 (*M*_*Women*_ = 31.91, *SD*_*Women*_ = 3.89; *M*_*Men*_ = 34.19, *SD*_*Men*_ = 6.28) participated in this study from the 27^th^ week of pregnancy until 40 weeks after birth. The majority of participants were Swiss (Women: 82.4%, Men: 81.3%). Relationship length ranged from 1.58 to 20.33 years (*M* = 7.13, *SD* = 4.06), and just over half of the couples (55.6%) were married at T1. The sample was well educated with 74.2% of all participants having a university degree. On average, couples earned between 60,000 and 80,000 US dollars per annum, which corresponds with the average salary of Swiss citizens [51]. We used all 104 couples throughout the analyses using a Full Information Maximum Likelihood (FIML) approach for the dropped out participants and missing data at each time point [52].

### Measures

#### Depressive symptomatology

Depressive symptoms were measured with the seven items of the depression subscale of the 21-item short form Depression-Anxiety-Stress Scale (DASS; [53]), which assesses the experience of depressive symptoms in the past week on a four point scale from 0 (*did not apply to me at all*) to 3 (*applied to me very much*, *or most of the time)*. There are cut-offs for clinically relevant levels of depression available for the DASS short-version, which involve doubling the sum of item scores (the original DASS was 42 items and scores for any of the 21-itme subscales are doubled for comparison with the 42 items original measure). Values between 10 and 13 indicate mild depressive symptomatology, 14 to 20 moderate, and over 21 indicate severe depressive symptomatology. In the current study, Cronbach’s alpha was high for women (T1: α = .93, T2: α = .81, T3: α = .89. T4: α = .83, T5: α = .80) and marginal to acceptable for men (T1: α = .79, T2: α = .55, T3: α = .72, T4: α = .78, T5: α = .75). Due to technical problems, three items of the DASS were not shown to about half of all male partners before birth, which likely affected reliability.

#### Dyadic coping

Dyadic coping was measured with the Dyadic Coping Inventory (DCI; [54]). The DCI is an instrument with 37 items assessing dyadic coping in couples with self- and partner-ratings. In the self-rating each partner is asked to rate the frequency of his or her own provided dyadic coping (DCpr) when the partner is stressed (e.g., ‘I show empathy and understanding to my partner’). In the partner-rating each partner is asked to rate the frequency of dyadic coping he or she receives from the partner (DCre) when feeling stressed (e.g., ‘My partner takes on things that I normally do in order to help me out’). The frequency of dyadic coping is rated on a 5-point Likert scale from 1 (*never*) to, 5 (*very often*). In the current study, reliability was good for women’s rating of provided (DCpr_w_ T1: α = .81, T2: α = .77, T3: α = .77, T4: α = .82, T5: α = .82), and received dyadic coping (DCre_w_ T1: α = .85, T2: α = .83, T3: α = .83, T4: α = .84, T5: α = .81) as well as for men’s ratings of provided (DCpr_m_ T1: α = .83, T2: α = .80, T3: α = .86, T4: α = .84, T5: α = .85), and received dyadic coping (DCre_m_ T1: α = .82, T2: α = .86, T3: α = .86, T4: α = .87, T5: α = .87). To control of the effect of dyadic coping itself, the average between provided and received dyadic coping as perceived by each partner was used as a predictor. By doing so, we were able to differentiate between couples with high or low overall frequency of dyadic coping and account for effects of equity of dyadic coping aside from the effects of dyadic coping itself [55].

#### Perceived equity of dyadic coping

Perceived equity of dyadic coping was measured using difference scores for each partner separately as proposed by the test manual [54] and experts in the analysis of dyadic data in cases when differences between partners’ overall levels are of interest (rather than similarity or congruence on each item) [56]. The perceived equity index reflected the difference for each partner between the dyadic coping they received and dyadic coping they provided. Provided coping (DCpr) was always subtracted from received coping (DCre) for equity perceived by women (EQU_w_ = DCrew—DCpr_w_) and men (EQU_m_ = DCrem—DCpr_m_). Accordingly, negative values indicated an underbenefit (received < provided) and positive values indicated an overbenefit (received > provided). Differences of zero represented the highest possible equity score. The more the values deviated from zero the lower the perceived equity. Because we used separate scores for men and women’s perceived equity, their perceptions could differ. For example, if Max rated the dyadic coping he received from Fiona with a 5 (DCre_m_ = 5) and the dyadic coping he provided with a 4 (DCpr_m_ = 4), he would have gotten a relatively high equity score (i.e., closer to zero) of 1 (EQU_m_ = 5–4 = 1). At the same time, Fiona could have rated the dyadic coping she received from Max with a 1 (DCre_w_ = 1) and the dyadic coping she provided with 3 (DCpr_w_ = 3), indicating an underbenefit (DCre_w_ < DCpr_w_) further away from zero than Max’s equity score (EQU_w_ = 1–3 = -2) and therefore a lower perceived equity than Maxes perceived equity score. Equity scores were independent from dyadic coping itself. For example, two couples could show perfect equity (0) on all indices while one couple showed a low frequency of dyadic coping (e.g., DCpr_m_ = 1, DCpr_w_ = 1, DCre_m_ = 1, DCre_w_ = 1) and the other couple showed a high frequency of dyadic coping (e.g., DCpr_m_ = 5, DCpr_w_ = 5, DCre_m_ = 5, DCre_w_ = 5). In order to analyze changes in the size of perceived equity, we further created absolute differences (distance scores), which lack indication of direction. To examine whether equity scores were associated with depressive symptoms in a linear or curvilinear manner, we squared difference scores as quadratic predictors additionally to the difference scores as linear predictors.

### Statistical analysis

To account for the longitudinal and dyadic structure of the data, a series of gender-specific two-level multilevel models were used [57], with Level 1 representing multiple times of assessment nested within individuals, and level 2 representing individuals nested within couples (between-dyad). Double-random-intercept-and-slopes multilevel models were used for the analyses using the multilevel package Nonlinear Mixed-Effects Models (nlme) version 2.6 [58] in R version 3.3.1 [59] using R Studio version 0.99.903, which provided separate estimates of intercept and slope for each gender. All variables were time-varying and correlated per time point. In the present study, fixed effects were of interest. While random variability of individuals across time was allowed in the models, only fixed effects will be reported. The nlme-package allows dealing with missing data by using the Full-Information-Maximum-Likelihood (FIML)-method, which estimates parameters for missing values without data imputation [52].

For the analysis of gender differences, we described the equity scores as perceived by men and women and conducted paired t-Tests. To analyze the effect of time on perceived equity of dyadic coping, growth curve models were calculated for men and women separately [60]. Because we expected different patterns of change (i.e., different growth rates) before and after birth, we used piecewise growth curve analyses with different predictors for time before birth and time after birth [61]. Change before birth (i.e., growth rate during third trimester of pregnancy) was analyzed with a time predictor of change in weeks before birth (TBB) from the 27^th^ week of pregnancy (TBB1: -5) to the 32^nd^ week of pregnancy (TBB2: 0) and time points after birth set to zero (TBB3: 0, TTB4: 0, TTB5: 0). Change after birth (i.e., growth rates after TTP) was analyzed with a time predictor of change in weeks after birth (TAB) with time points before before birth set to zero (TAB1: 0, TAB2: 0) and time points after birth in weeks (TAB3: 2, TAB4: 14, TAB5: 40). To see if there is a change in the size of equity independent from direction, we used absolute distance scores obtained by absolute differences of women’s perceived equity (EQU_wa =_ |DCre_w_—DCpr_w_|) and men’s perceived equity (EQU_ma =_ | DCre_m_- DCpr_m_). For example, if DCre_m_ = 1 and DCpr_m_ = 5, the size of EQU_ma_ is |4|. Then, absolute difference scores were predicted by TBB and TBB. For example, an increase of Max’s equity score from |1| to |4| would tell us that the equity decreased over time (further away from 0). In a second step, we were interested in changes towards over- or underbenefit of women and men. Therefore, difference scores were predicted by time., For example, a change in Maxes equity score from -1 to -4 would indicate an increasing underbenefit and a change from 1 to 4 an increasing overbenefit over time.

For our third research question we focused on the associations between equity of dyadic coping and depressive symptoms. To disentangle the effect of the equity from the effect of dyadic coping itself, we also used the level of dyadic coping itself as a predictor [55]. Time was added as a control variable. We used person-level predictors and outcomes of depressive symptoms for men and women separately, as it made sense theoretically that men and (pregnant) women might differ significantly in their equity perceptions and depressive symptomatology. Model comparisons were made starting from the simplest model with only random intercepts (model 0). Next, the control variable time was added as predictor (model 1), then dyadic coping (model 2), linear equity predictors (model 3), quadratic equity predictors (model 4), partner effects of the equity predictors (model 5), and the interaction between equity of dyadic coping and dyadic coping itself (model 6).

## Results

### Descriptive statistics

From the 104 couples used for the current analyses, 93 couples (88.6%) participated at all time points. AT T2, four couples dropped out (4.2%), at T3 three couples (3.0%), at T4 zero couples, and at T5 four couples (3.9%). Couples who dropped out did not differ in their nationality, relationship length, civil status, education, or income. However, women and men who dropped out were significantly older than those who did not drop out (women: *t*(102.25) = 82.27, *p* < .001; men: *t*(101.48) = 54.77, *p* < .001). Compared to women who did not drop out, women who dropped out during the course of the study showed higher depressive symptoms (*t*(101.58) = 9.54, *p* < .001), and higher perceived equity (*t*(202.33) = 6.08, *p* < .001) at T1. Compared to men who did not drop out, men who dropped out showed lower depressive symptoms (*t*(103.04) = 7.47, *p* < .001), and higher perceived equity (*t*(169.71) = 4.15, *p* < .01) at T1.

### Depression across the TTP

At the 27^th^ week of pregnancy, 27.3% of all women showed at least mild depressive symptomatology. Over time, the percentage of women with at least mild depressive symptomatology decreased, 21.8% at the 32^nd^ week, 21.1% at 2 week after birth, 8.7% at 14 weeks after birth, and 6.4% at 40 weeks after birth. The percentage of men with at least mild depressive symptomology was 14.1% during the 27^th^ week of pregnancy, 9.6% during the 32^nd^, and 4.4% at 2 weeks after birth, 8.1% at 14 weeks, and 12.0% at 40 weeks after birth. As shown in Table 1, women showed a decrease of depressive symptoms after birth while men’s depressive symptoms stayed relatively stable. Women showed higher depressive symptoms than men on both time points before birth and two weeks postpartum, however at 14 weeks and 40 weeks postpartum men showed higher depressive symptoms. Women and men’s depressive symptoms showed a small but significant intraclass correlation at T1 (*ICC*(1) = .041, *p* = .022). The intraclass correlations within couples were not significant at all other time points.

**Table 1:** Descriptive statistics of women and men’s depressive symptoms and perceived equity of dyadic coping across TTP.

	T1		T2		T3		T4		T5		TBB	TAB
Main variables	M (SD)	Range	M (SD)	Range	M (SD)	Range	M (SD)	Range	M (SD)	Range	*β*	*β*
Women’s Depression	3.37 (3.42)	0–21	2.85 (2.13)	0–9	3.57 (3.36)	0–15	1.84 (2.37)	0–11	1.45 (2.28)	0–13	-0.01	-0.05***
Men’s Depression	1.96 (2.47)	0–12	2.02 (2.06)	0–7	1.58 (2.41)	0–16	1.94 (2.81)	0–17	1.98 (2.63)	0–13	-0.02	-0.00
*t*	5.07***		3.62***		6.37***		-0.70		-3.25**			
Dyadic Coping (DC)												
Provided by women	4.23 (0.43)	3–5	4.26 (0.35)	3–5	4.22 (0.36)	3–5	4.17 (0.41)	3–5	4.11 (0.41)	2–5	-0.00	-0.00***
Provided by men	4.02 (0.43)	3–5	3.96 (0.42)	3–5	4.00 (0.48)	3–5	3.91 (0.49)	3–5	3.88 (0.50)	3–5	-0.01^(*)^	-0.00*
*t*	6.20***		6.35***		5.07***		5.74***		4.34***			
Received by women	3.95 (0.51)	3–5	4.00 (0.46)	3–5	3.93 (0.48)	3–5	3.87 (0.49)	2–5	3.76 (0.50)	2–5	0.00	-0.00***
Received by men	4.10 (0.43)	3–5	4.07 (0.46)	3–5	4.06 (0.47)	3–5	4.03 (0.51)	2–5	3.96 (0.51)	3–5	-0.01	-0.00*
*t*	-3.64***		-2.45*		-2.38*		-2.61**		-4.34***			
Women’s perceived equity of dyadic coping	-0.29 (0.35)	-1.2 - .5	-0.26 (0.36)	-1.4–0.5	-0.3 0(0.35)	1.6–0.4	-0.30 (0.42)	-.87–0.6	-0.35(0.40)	-1.9–0.5	0.00	0.00^(*)^
Men’s perceived equity of dyadic coping	0.08 (0.30)	-0.7–0.1	0.11 (0.29)	-0.7–0.7	0.06 (0.31)	-0.9–1.0	0.12 (0.34)	-.60–1.0	0.08 (0.32)	-1.1–0.7	0.00	0.00
*t*	-10.65***		-9.52***		-9.30***		-8.85***		-9.77***			

T1 = 27^th^ week of pregnancy, T2 = 32nd week of pregnancy, T3 = two weeks postpartum, T4 = 14 weeks postpartum, T5 = 40 weeks postpartum, TBB = time before birth, TAB = time after birth

(*) p < .100

* p < .050

** p < .010

*** p < .001.

### Gender differences in perceived equity of dyadic coping

Descriptive statistics depicted in Table 1 showed significant differences between men and women at all time points. Women provided significantly more dyadic coping than their male partners at all time points. Furthermore, women received less dyadic coping than men received at all time points. Accordingly, with regard to perceived equity, women showed negative scores and men showed positive scores at all time points. Thus, women perceived that they received less dyadic coping than they provided (underbenefit) and men perceived that they received more dyadic coping than they provided (overbenefit). While men and women seemed to agree that women provided more dyadic coping than they received, women evaluated the perceived equity as significantly lower than men at all time points. Thus, men perceived lower differences between provided and received dyadic coping than women.

Bivariate correlations showed that perceived equity scores of men and women correlated significantly at T5 only, when men and women’s equity showed a negative correlation (*r* = -.250, *p* < .001), indicating that men’s underbenefit was associated with women’s overbenefit and vice versa (marginal associations in the same direction were visible at T1 (*r* = -.133, *p* = .060) and T4 (*r* = -.153, *p* = .066)). Dyadic coping itself showed no significant associations with men’s perceived equity at any time point. In women, dyadic coping itself showed significant associations with women’s perceived equity at all time points, indicating that overbenefits were associated with more dyadic coping and underbenefits with less dyadic coping (T1: *r* = .334, *p* < .001; T2: *r* = .358, *p* < .001; T3: *r* = .364, *p* < .001; T4: *r* = .226, *p* = .039; T5: *r* = .238, *p* = .024) and correlations with distance scores showed that higher perceived equity (lower absolute difference) in women was associated with more dyadic coping (T1: *r* = -.272, *p* = .006; T2: *r* = -.418 *p* < .001; T3: *r* = -.377 *p* < .001; T4: *r* = .320, *p* = .003; T5: *r* = -.314, *p* = .003). The correlations indicate that perceived equity is moderately associated with but not identical to dyadic coping itself.

### Perceived equity across the TTP

In our first hypothesis, we assumed that perceived equity decreases across the TTP. To test this hypothesis, we analyzed piecewise growth curve models for men and women separately. To detect changes in size of equity (independent from direction), we used absolute difference scores as outcomes. Predictors of change were weeks with different growth rates for time before and after birth.

**Fig 1:**
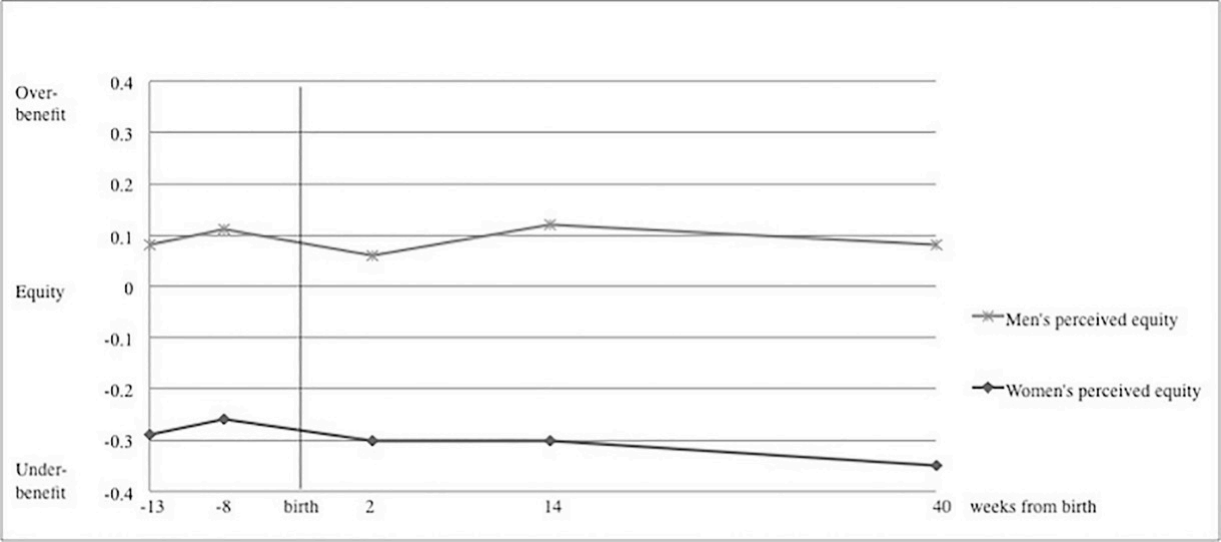
Perceived equity of dyadic coping across the TTP.

Descriptively, perceived equity scores in women increased after birth (see Fig 1), which means that differences became larger (further away from zero) over time and therefore women’s equity became lower over time. Men’s perceived equity stayed relatively stable or even seemed to increase over time. The growth curve analyses revealed no significant changes in equity as perceived by men. Women’s perceived equity showed a marginal decrease (*β* = .001, SE = 0.001, *t* = 1.75, *p* = .081) when using absolute differences as outcomes. When using difference scores as outcomes, no statistically significant changes in either direction of women or men’s benefit was visible.

### Perceived equity of dyadic coping and depressive symptoms

Our third research question addressed the association between perceived equity and depressive symptoms across the TTP. To analyze this association a random-intercept-and-slopes model with one intercept per partner was used to obtain effects for men and women separately controlling for partners’ interdependence as well as interdependence of person’s values over time. Upon model comparison model 5 fitted the data best. We therefore report results from model 5 here, estimates from all other models can be found in the supplementary material. We controlled for the effect of time before and after birth. In women, depressive symptoms decreased after birth. No significant changes were found before birth or after birth in men (see Table 2).

**Table 2:** Associations between depressive symptoms and equity of dyadic coping across the TTP.

	Women				Men			
	*β*	*SE*	*T*	*P*	*β*	*SE*	*t*	*p*
**Intercept**	2.90	0.25	11.65	< .001	1.56	0.25	6.34	< .011
**Time before birth**	-0.00	0.05	-0.04	.967	-0.02	0.05	-0.45	.651
**Time after birth**	-0.05	0.01	-6.41	< .001	-0.00	0.01	-0.44	.656
**Equity (actor)**	0.42	0.54	0.78	.435	0.64	0.43	1.50	.134
**Equity Q (actor)**	1.60	0.49	3.29	.001	0.77	0.80	0.97	.333
**Equity (partner)**	-0.26	0.41	-0.62	.534	0.17	0.57	0.30	.760
**Equity Q (partner)**	0.55	0.76	0.72	.472	1.32	0.50	2.64	.008
**Dyadic Coping**	-0.60	-0.36	-1.66	.098	-1.05	0.34	-3.81	.002

Equity = perceived equity of dyadic coping by men or women separately, actor = actor effect (women’s equity on women’s depression, men’s equity on men’s depression). Q = quadratic predictors for curvilinear associations, partner = partner effect (women’s equity on men’s depressive symptoms, men’s equity on women’s depressive symptoms).

**Fig 2:**
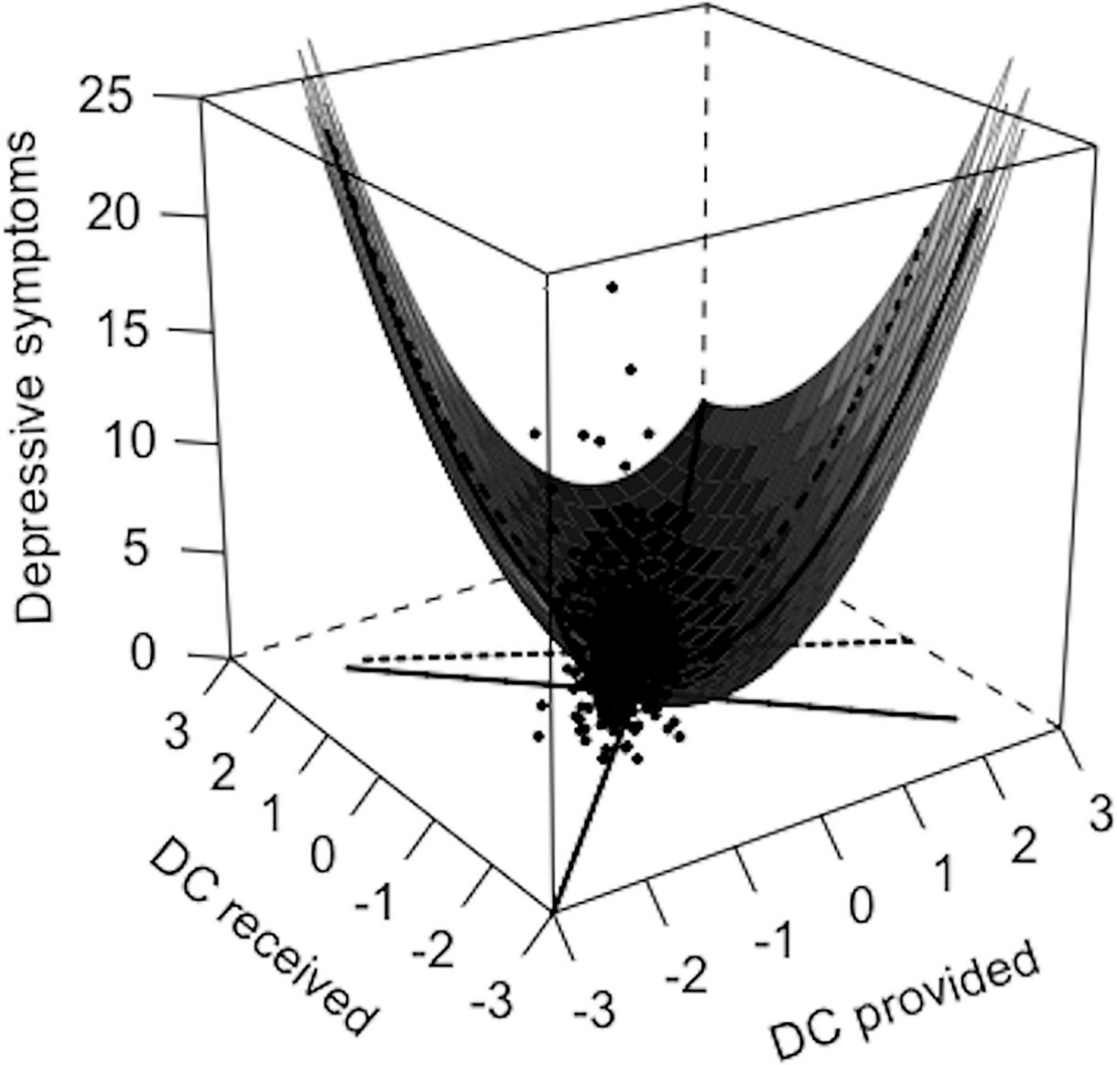
Regressions of women’s depressive symptoms on women’s perceived equity. *Note*. DC = dyadic coping. Significant curvilinear association between women’s equity and women’s depressive symptoms (actor effect) as visible by lower depressive symptoms along the line of equity (front to back corner) where provided and received dyadic coping are perceived to be the same. Linear associations were not significant as visible by higher depressive symptoms in either direction of inequity, i.e., higher depressive symptoms in the case of overbenefit (received > provided DC; left corner) or underbenefit (received < provided DC; right corner).

**Fig 3:**
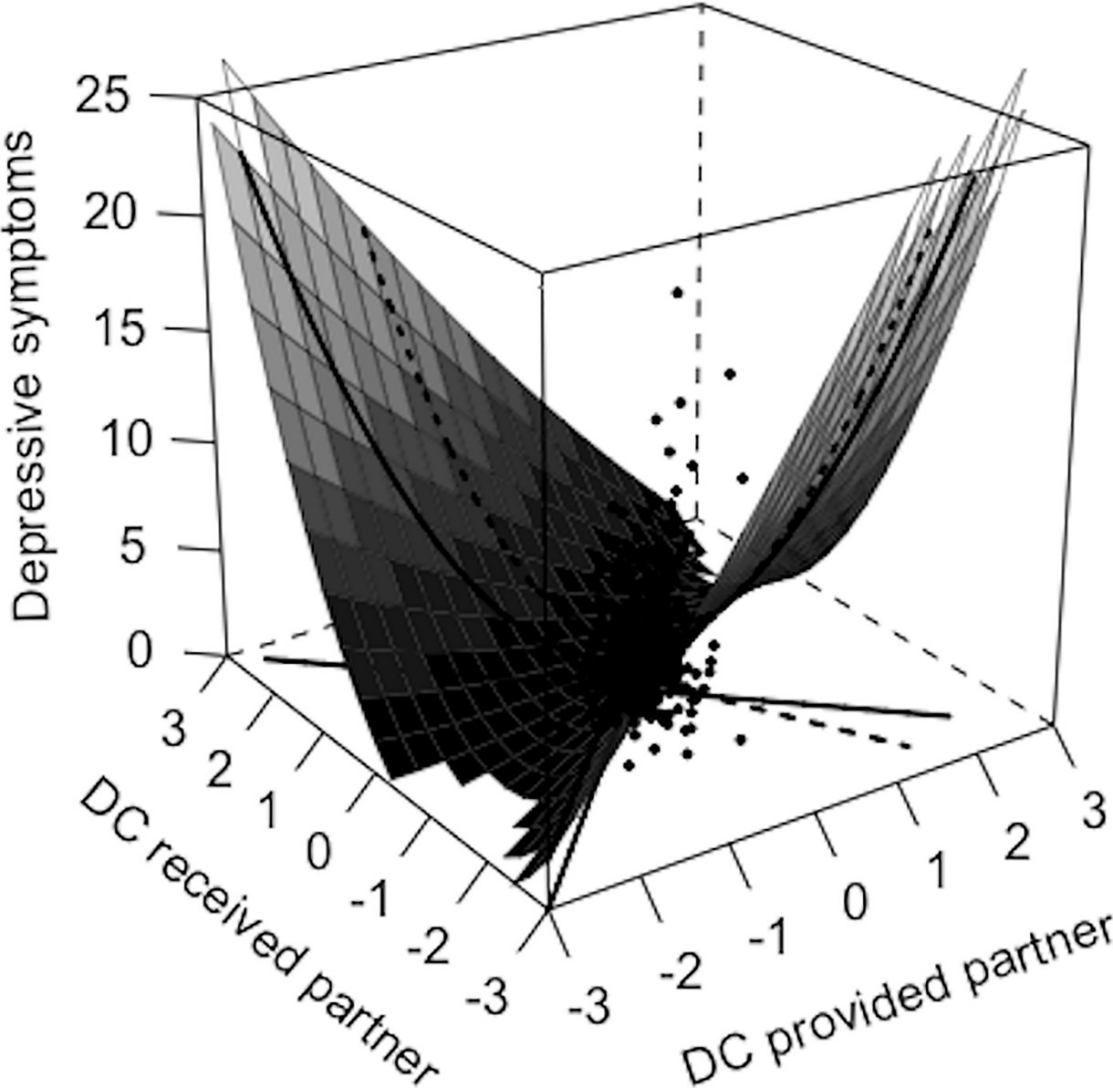
Regression of men’s depressive symptoms on women’s perceived equity (partner effects). *Note*. DC = dyadic coping. Significant curvilinear association between women’s equity and men’s depressive symptoms (partner effect) as visible by lower depressive symptoms in men along the line of equity as perceived by their female partners (front to back corner) where provided and received dyadic coping are perceived to be the same. Linear associations were not significant as visible by higher depressive symptoms in either direction of inequity (i.e., in the case of overbenefit (received > provided DC; left corner) or underbenefit (received < provided DC; right corner)).

We expected a curvilinear association between equity of dyadic coping and depressive symptoms above and beyond the effect of the dyadic coping itself. Accordingly, the quadratic equity predictor showed significant associations with depressive symptoms in women, while the linear predictor showed no significant associations in women or men. As shown in Fig 2, women’s depressive symptoms were low on the line of equity (front to back line) when women perceived the dyadic coping they received to be similar to the dyadic coping they provided. Women’s depressive symptoms were higher in both directions of inequity independent whether women perceived an overbenefit (left corner) or underbenefit (right corner). In men, depressive symptoms were not associated with men’s perceived equity. In a second step, we checked for partner effects. While women’s depressive symptoms were not associated with men’s perceived equity, we found partner effects for men. As visible in Fig 3, men showed lower depressive symptoms when their female partners perceived a high equity of dyadic coping (line of equity from front to back corner as perceived by female partners) and higher depressive symptoms when women perceived a lower equity of dyadic coping in either direction (overbenefit: left corner; underbenefit: right corner). Linear predictors showed no significant associations in women or men.

The associations between perceived equity of dyadic coping and depressive symptoms were controlled for dyadic coping itself. We found the expected negative associations between dyadic coping itself and depressive symptoms in men but only marginal negative associations for women’s depressive symptoms (see Table 2). We found no significant moderation of dyadic coping itself on the association between equity and depressive symptoms for women (*β* = 1.04, SE = 0.85, *t* = 1.22, *p* = .224) or men (*β* = -0.69, SE = 0.88, *t* = 0.78, *p* = .433). The model that included the interaction term as a predictor did not fit better (AIC = 3822.28, BIC = 3958.89, logLik = -1882.14) than the model without the interaction term (AIC = 3821.04, BIC = 3948.29, logLik = -1883.53) upon model comparison (*p* = .252).

## Discussion

This paper explored equity of dyadic coping across the TTP of mixed-gender couples. As gender roles become more traditional in domestic tasks [62] and women’s domestic work load tends to increase while men’s does not [63], we expected such a shift towards women providing increasingly more dyadic coping compared to men across the TTP. In line with our assumptions, we found that women provided more and received less dyadic coping than men at all time points. While we also find gender differences in dyadic coping in community samples [31], they tend to be rather small [42]. In couples becoming parents, they seemed to exacerbate and persevere, possibly also due to a high level of stress [64]. While men and women both perceived a gender difference in the direction of underbenefit for women, men perceived a higher equity on average than women did at all time points. We do not know, whether men overestimated or women underestimated the equity of dyadic coping across TTP. As dyadic coping is introduced as ‘support when the partner is stressed’ it is a rather context-unspecific behavior and is therefore susceptible to perceptive biases [65]. Furthermore, the TTP is an emotional sensitive phase, which makes perceptive biases likely [66]. It is likely that parents did not expect such a decrease of dyadic coping across TTP and then projected their own decreased coping provision onto the partner [42]. To examine which perception is closer to the actual reality, more direct or behavioral measures would be important. However, previous studies found the subjective perception of equity to be more important for couples’ adaption than actual similarity of inputs and outputs [67].

While we found that women’s perceived equity tended to decrease across TTP, men’s perceived equity remained stable. Even though the small but descriptively visible decreases in perceived equity might have been statistically significant in a larger sample with more variance (overall perceived equity scores were rather close to zero in all couples) we did expect to find a stronger decrease in women’s equity and at least moderate decreases in men’s perceived equity. However, it is again consistent with literature on the traditionalization of gender roles across the TTP [14] that changes in perceived fairness mostly affect women [62]. Furthermore, men were found to show surprising stability in other variables such self-control [68] or housework [69] across the TTP, in contrast to considerable changes in women.

Some might argue that women are more vulnerable to changes of TTP and are more distressed by it, as they are the ones who are pregnant, giving birth, and often times breast feeding and taking care of the infant after birth, and therefore they will need to receive more dyadic coping than they provide. Indeed, we found significantly more depressive symptoms in women before and right after birth. However, this difference disappeared after 14 weeks postpartum (the time when the mandatory maternity leave ends in Switzerland). At 40 weeks postpartum, men even showed more depressive symptoms. Furthermore, independent from these differences, women’s perceived equity showed associations with depressive symptoms at all time points. Additionally, we found no indication that receiving more dyadic coping than providing was associated with less depressive symptoms. In contrast, we only found curvilinear associations between equity and depressive symptoms. These findings are in line with the equity theory [70] and in contrast to the cost-optimizing assumptions of the social exchange theory [44]. It seems to be more important for women’s mental health to provide similar amounts of dyadic coping as they receive. Accordingly, feeling advantaged in the relationship was associated with more anxiety in women before birth [15] and perceived inequity in infant care was associated with more depressive symptoms across the TTP [11]. Nonetheless, we did have more variance and a higher severity of depressive symptoms for women in our sample. A bidirectional association cannot be precluded, insofar as more depressed women would be less likely to provide a lot of support [71]. Especially in women with high levels of depression, lower provided dyadic coping was found [72]. Furthermore, individuals with depression show impairments in their perception of other’s behaviors [73]. It is possible that parents with more depressive symptoms underestimated support received by the partner due to cognitive impairments. However, while we did not ask how fair partners perceive the distribution of dyadic coping within the couple, previous studies found the subjective perception of equity to be more important for couples’ adaption than actual equity [67].

Another contra point to the assumptions of the equity theory could be that women need more support during the TTP and both members of the couple would conclude that it is fair for a certain period of time that women receive more support in times of stress than men. Additionally, male partners might even feel a higher sense of control [74] and higher self-esteem when providing more support than receiving [75]. However, our results point to the opposite. While men’s perceive equity was not associated with their depressive symptoms, they showed lower depressive symptoms when their female partners perceived a high equity of dyadic coping. An overbenefit seems not to be beneficial for woman across TTP and neither is providing more dyadic coping than receiving for men. Even more so, women were found to be more distressed by feeling generally overbenefitted than underbenefitted in the relationship [15].

Last but not least, equity of dyadic coping and dyadic coping itself seem to be independent predictors of depression across TTP. We did not find an interaction effect. Dyadic coping itself was negatively associated with depressive symptoms in men and marginally significant in women. Men’s perceived equity showed no significant association with depressive symptoms. Women’s perceived equity, on the other hand, showed the largest associations with depressive symptoms in women and men, beyond dyadic coping itself. The beneficial effect of couples’ dyadic coping has been shown in various studies [49,50], but the role of equity of dyadic coping has been understudied. Many questions remain to be answered. For example, we might have found a significant interaction with a larger sample. However, model fits pointed to the contrary. We can only assume potential mechanisms between equity and depressive symptoms such as sense of control [74], relationship satisfaction, or self-esteem [75]. Nonetheless, small but robust gender-specific associations between equity and depressive symptoms indicate an independent contribution of equity in the ‘relationship sphere’ on individual adjustment.

### Strength and limitations

The study design was strong with five times of measurement as well as self- and partner-ratings of men and women across the TTP. The results were obtained using state of the art dyadic data analyses and innovative examination of different aspects of equity. Despite a rich data sample, a few limitations of this study need to be mentioned. First, the sample consisted of volunteers for a TTP education trial who were assigned to the control group. As such, couples were well-educated and in a stable relationship; both are protective factors against depression [76–78], and so the generalizability of the results is limited. The lower equity in couples that dropped out could be an indicator of a selective sample. Additionally, this was a non-clinical sample. Few participants met the clinical cut-off for severe depression. Furthermore, depressive symptoms decreased in our study. We can only speculate whether this was due to our specific sample or if depressive symptoms have already increased during pregnancy. On the other hand, decreases in psychopathology were found in earlier studies [8]. Further studies are needed to test whether effects are even stronger in (sub-)clinical samples. Furthermore, the range of the equity indices was rather small. This seems to indicate that couples perceive the support they provide and receive as being rather equitable, also across TTP. This may result from a high level of overall equity or from a positive bias of couples becoming parents. To deepen our understanding of couples’ perceptions of equity across TTP, future studies should use samples with more variance (e.g., higher risk) and measures with fewer biases (e.g., observational data). Additionally, we can assume that depressive symptoms had already started to rise during pregnancy, as suggested by literature [79], which would in turn explain the decrease of depressive symptoms in our sample. It would be interesting to include couples earlier during pregnancy or to be able to include baseline values for depressive symptomatology before pregnancy–although this might be difficult to achieve for practical reasons. Finally, yet importantly, we used difference scores as measurements of equity, because we were innately interested in the differences themselves. While the use of difference scores was proposed by dyadic data experts [56] and further developed into dyadic score models [55], there was also some criticism on the use of difference scores in the past [80,81]. While first, this criticism was particularly focused on individual change scores rather dyadic data and second, we countered these critiques by controlling for the level of dyadic coping, the use of various times of measurements, satisfying reliability, and variability over time, there are some promising recent developments of alternative methods to assess equity in dyadic data [74,75], which might eventually be suitable to use for the comparison of actor and partner effect within a longitudinal setting. Nonetheless, adding direct and observed fairness measures to the measures of equity could also shed more light on this important and complex phenomenon. Future research should explore mechanisms as well as the interplay between equity in the domestic sphere and equity of dyadic coping, the role of cultural and political contexts, and if and when equity can be gradually restored as children grow older.

### Clinical implications

When couples become parents they face a wide array of stressors, which can affect their mental health. Because depressive symptomatology is interdependent in romantic partners [82], not only couple counselors but also public health practitioners should include both partners into screening procedures and psycho-education of mental health across TTP [83]. To take on a systemic transactional perspective on mental health does not only mean that both partners are stressed, but that both partners bring along resources to cope with these stressors as well [84]. It is therefore an important task for couples and couple counselors to foster couples’ joint coping skills (i.e., dyadic coping). Additionally, our results indicate that differences in the perceptions of both partners should be measured and addressed in therapy. Perceived inequity of dyadic coping as well as expectations connected to supporting one another should be made explicit, be discussed, and reduced–ideally already before the TTP. For example, relationship education programs addressing normative changes across TTP might reduce disappointed expectations and perceived loss of control [85].

## Conclusions

This study aims to add the important puzzle piece that is perceived equity of dyadic coping to our knowledge of the TTP. This puzzle piece intends to help couples that cope with the stress of parenthood and to prevent them from disappointed expectations, diverging perceptions, and a rise of distress. Albeit the emerging egalitarianism in Western cultures, parenthood seems to remain gendered [86]. We must remain sensitive to gender differences, as inequity between men and women is associated with lower health in both, may it be in the domestic [87] or relationship sphere. We might want to go beyond mother and child onto a relationship perspective, taking into account both partners’–potentially very different–perceptions of one and the same phenomenon. This study has shown that the difference between provided and received dyadic coping is meaningful. How equal both partners supported each other contributed to their adjustment independently from how well they supported each other across TTP.

## Supporting information

S1 File Estimates and fit indices of all tested models. (PDF)
