# Supplementary material for: Fair enough? Decreased equity of dyadic coping across the transition to parenthood associated with depression of first-time parents
Source: PLoS One. 2020 Feb 19;15(2):e0227342. doi: 10.1371/journal.pone.0227342 (PMC7029854; doi:10.1371/journal.pone.0227342)
Supplement: S1 File — (PDF) [file pone.0227342.s001.pdf]

S1 File. Estimates and fit indices of all tested models.

Model 0: Depressive symptoms random-intercepts model.

	Women					Men			
	β	***SE***	***t***	***p***		***β***	***SE***	***t***	***p***
**Intercept**	2.30	0.18	12.65	.000		1.91	0.19	10.11	.000

Note. Model fit indices: AIC = 4197.63, BIC = 4259.97, logLik = -2085.82.

Model 1: Depressive symptoms predicted by time.

	Women					Men			
	β	***SE***	***t***	***p***		***β***	***SE***	***t***	***p***
**Intercept**	3.20	0.24	12.11	<.001		1.93	0.22	8.83	<.001
**Time before birth**	-0.03	0.05	-0.64	.524		-0.02	0.05	-0.32	.752
**Time after birth**	-0.05	0.01	-5.91	<.001		0.00	0.01	0.25	.799

Note. Model fit indices: AIC = 4191.99, BIC = 4273.44, logLik = -2078.99.

Model 2: Depressive symptoms predicted by time and dyadic coping.

	Women					Men			
	β	*SE*	*t*	*p*		*β*	*SE*	*t*	*p*
**Intercept**	3.34	0.24	14.12	<.001		1.94	0.21	9.11	<.001
**Time before birth**	-0.03	0.05	-6.57	.545		-0.03	0.05	-0.53	.594
**Time after birth**	-0.05	0.01	-6.57	<.001		-0.00	0.01	-0.15	.884
**Dyadic Coping**	-1.52	0.35	-4.32	<.001		-1.17	0.33	-3.59	<.001

Note. Model fit indices: AIC = 4147.21, BIC = 4238.07, logLik = -2054.60.

Model 3: Depressive symptoms predicted by time, dyadic coping, and linear equity predictors.

	Women					Men			
	β	*SE*	*t*	*p*		*β*	*SE*	*t*	*p*
**Intercept**	3.16	0.26	12.19	<.001		1.86	0.21	8.71	<.001
**Time before birth**	-0.03	0.05	-0.58	.561		-0.03	0.05	-0.58	.563
**Time after birth**	-0.05	0.01	-6.62	<.001		-0.00	0.01	-0.15	.881
**Equity (actor)**	-0.62	0.36	-1.72	.086		0.88	0.41	2.15	.032

**Dyadic Coping**	-1.38	0.36	-3.81	<.001		-1.23	0.33	-3.78	<.001

Note. Model fit indices: AIC = 4144.54, BIC = 4244.92, logLik = -2051.27.

Model 4: Depressive symptoms predicted by time, dyadic coping, linear, and quadratic equity predictors.

	**Women**					**Men**			
	β	***SE***	***t***	***p***		***β***	***SE***	***t***	***p***
**Intercept**	3.16	0.26	12.29	<.001		1.77	0.23	7.82	<.001
**Time before birth**	-0.03	0.05	-0.65	.512		-0.03	0.05	-0.57	.567
**Time after birth**	-0.05	0.01	-6.67	<.001		-0.00	0.01	-0.20	.840
**Equity (actor)**	0.20	0.55	0.36	.717		0.73	0.43	1.69	.090
**Equity Q (actor)**	0.99	0.50	1.98	.048		1.02	0.82	1.25	.210
**Dyadic Coping**	-1.27	0.36	-3.49	<.001		-1.16	0.33	-3.52	<.001

Note. Model fit indices: AIC = 4141.54, BIC = 4251.43, logLik = -2047.77.

Model 5: Depressive symptoms predicted by time, dyadic coping, linear, and quadratic equity predictors with actor and partner effects.

	**Women**					**Men**			
	β	***SE***	***t***	***p***		***β***	***SE***	***t***	***p***
**Intercept**	2.90	0.25	11.65	<.001		1.56	0.25	6.34	<.001
**Time before birth**	-0.00	0.05	-0.04	.967		-0.02	0.05	-0.45	.651
**Time after birth**	-0.05	0.01	-6.41	<.001		-0.00	0.01	-0.44	.656
**Equity (actor)**	0.42	0.54	0.78	.435		0.64	0.43	1.50	.134
**Equity Q (actor)**	1.60	0.49	3.29	.001		0.77	0.80	0.97	.332
**Equity (partner)**	-0.26	0.41	-0.62	.534		0.17	0.57	0.30	.761
**Equity Q (partner)**	0.55	0.76	0.76	.472		1.32	0.49	2.64	.008
**Dyadic Coping**	0.59	0.36	-1.66	.098		-1.05	0.34	-3.08	.002

Note. Model fit indices: AIC = 3821.04, BIC = 3948.29, logLik = -1883.52.

Model 6: Depressive symptoms predicted by time, dyadic coping, linear, and quadratic equity predictors with actor and partner effects, and the interaction of dyadic coping and equity

	**Women**					**Men**			
	β	***SE***	***t***	***p***		***β***	***SE***	***t***	***p***
**Intercept**	2.88	0.25	11.63	<.001		1.57	0.25	6.30	<.001
**Time before birth**	-0.00	0.05	-0.06	.949		-0.02	0.05	-0.44	.661
**Time after birth**	-0.05	0.01	-6.41	<.001		-0.00	0.01	-0.47	639
**Equity (actor)**	0.40	0.55	0.74	.462		0.627	0.44	1.41	.158
**Equity Q (actor)**	1.61	0.56	2.89	.004		0.79	0.80	0.98	.326
**Equity (partner)**	-0.28	0.41	-0.68	.497		0.17	0.57	0.30	.761
**Equity Q (partner)**	0.55	0.77	0.72	.474		1.32	0.50	2.61	.001
**Dyadic Coping**	-0.60	0.42	-1.43	-153		-1.04	0.34	-3.05	.002
**Interaction**	0.02	0.83	0.03	.976		-0.05	0.88	-0.06	.956

Note. Model fit indices: AIC = 3822.28, BIC = 3958.89, logLik = -1882.14.
